# Supplementary material for: Physics-based evolution of transmembrane helices reveals mechanisms of cholesterol attraction
Source: Nat Commun. 2025 Oct 20;16:9275. doi: 10.1038/s41467-025-63769-5 (PMC12537875; doi:10.1038/s41467-025-63769-5)
Supplement: Supplementary file 1 — Supplementary Information [file 41467_2025_63769_MOESM1_ESM.pdf]

Supplementary Information

Methods

Native epithelial membrane

A cholesterol attractor ($D_3K_3L_8K_3D_3$) was inserted into a native epithelial membrane model ((*1*), ”Membrane 1”) and a 2 μs NPT MD simulation is performed with a 20 fs time step, of which the first 500 ns were used for equilibration purposes. Temperature was coupled to 310 K using velocity rescaling ($\tau = 1$ ps, with separate coupling groups for the membrane, peptide, and solvent). Pressure was coupled semi-isotropically to 1 bar using the Parrinello-Rahman barostat ($\tau = 12$ ps), with compressibility set to $3.0 * 10^{-4}$ bar^{-1}.

Cholesterol content was computed from the ratio of cholesterol molecules to membrane molecules within a cylinder of radios r centered on the peptide center-of-mass (COM) (i.e. $f_{ratio}(r) = \frac{g_{CN,CHOL}(r)}{g_{CN,Lipids}(r)}$).

Restraining peptide transmembrane position

Several peptides with unfavorable transmembrane affinity have been restrained to better investigate the underlying effect of hydrophobic block length on cholesterol attraction. After a peptide structure was inserted into the template membrane, a short 5 ns relaxation step was performed to position the peptide. The first and last backbone beads of the peptide were then restrained using a flat-bottom potential to 0.25 nm thick layers parallel to the membrane, with respect to their initial relaxed positions. To prevent the membrane from adjusting to the peptides enforced position, all lipid beads were restrained to a 5.52 nm thick layer centered and parallel to the initial membrane configuration.

Free-energy calculations

To determine the transmembrane stability of peptides, we computed the free energy of insertion ($\Delta G_{insertion}$) using the thermodynamic integration (TI) method. For both flat and TM configurations, coulomb and Van der Waals interactions were decoupled separately in steps of $\lambda = 0.05$. The free energy then becomes $\Delta G_{insertion} = \Delta G_{flat,coul} + \Delta G_{flat,vdw} - \Delta G_{TM,vdw} - \Delta G_{TM,coul}$.

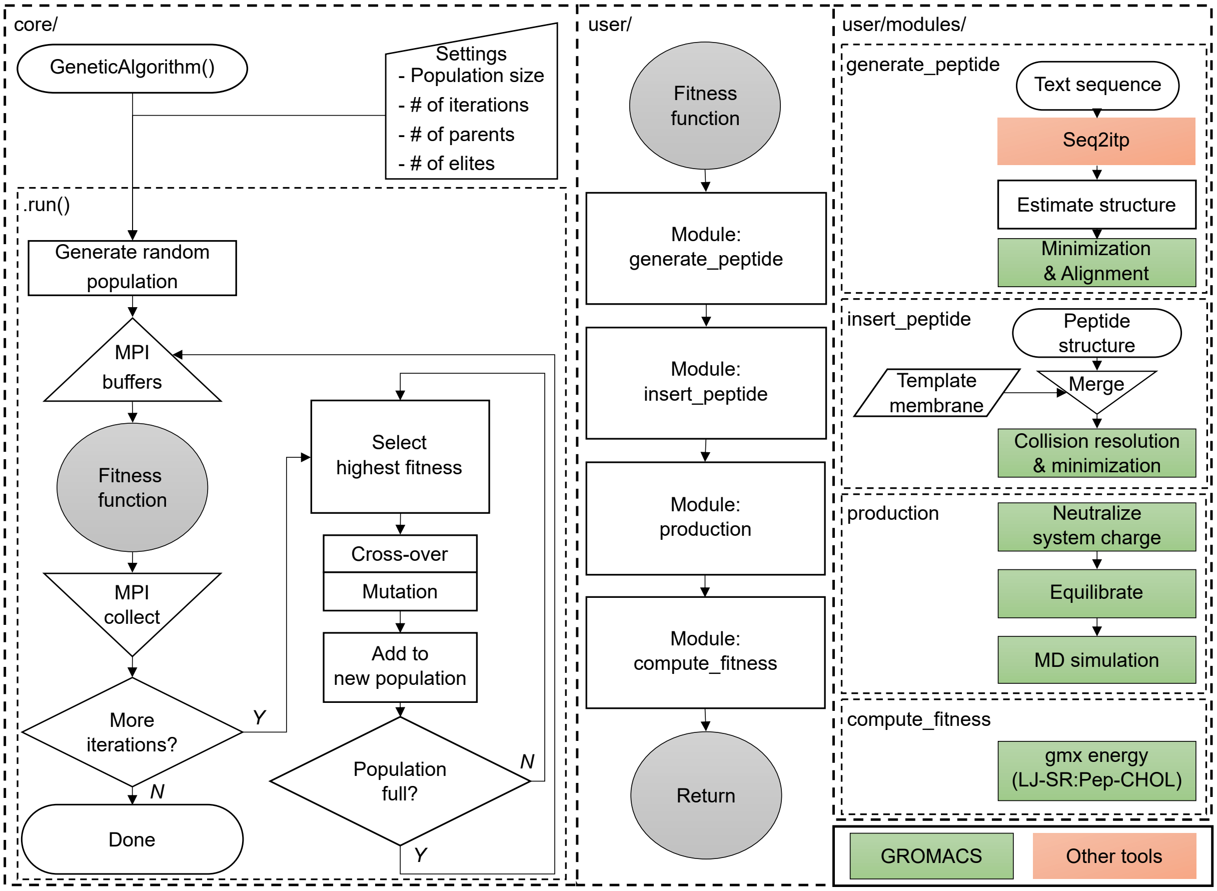

Supplementary Figure 1. **Flowchart of EVO-MDimplementation. (core/)** Genetic algorithm core of EVO-MD. An instance of the GeneticAlgorithm class is made and initialized with run parameters (e.g. population size, sequence length, etc...). The population is then initialized with random sequences and evaluated over all available MPI ranks using the fitness function. Best performing sequences are selected based on fitness, and a new population is formed using cross-over and mutation operators. **(user/ and user/modules/)** Cholesterol attractor implementation into EVO-MD. Fitness function receives a peptide sequence from the GA from which a simulation system is generated. Using the resulting simulation trajectory, a fitness value is computed based on non-bonded interaction energies between cholesterols and peptide, which is returned to the GA.

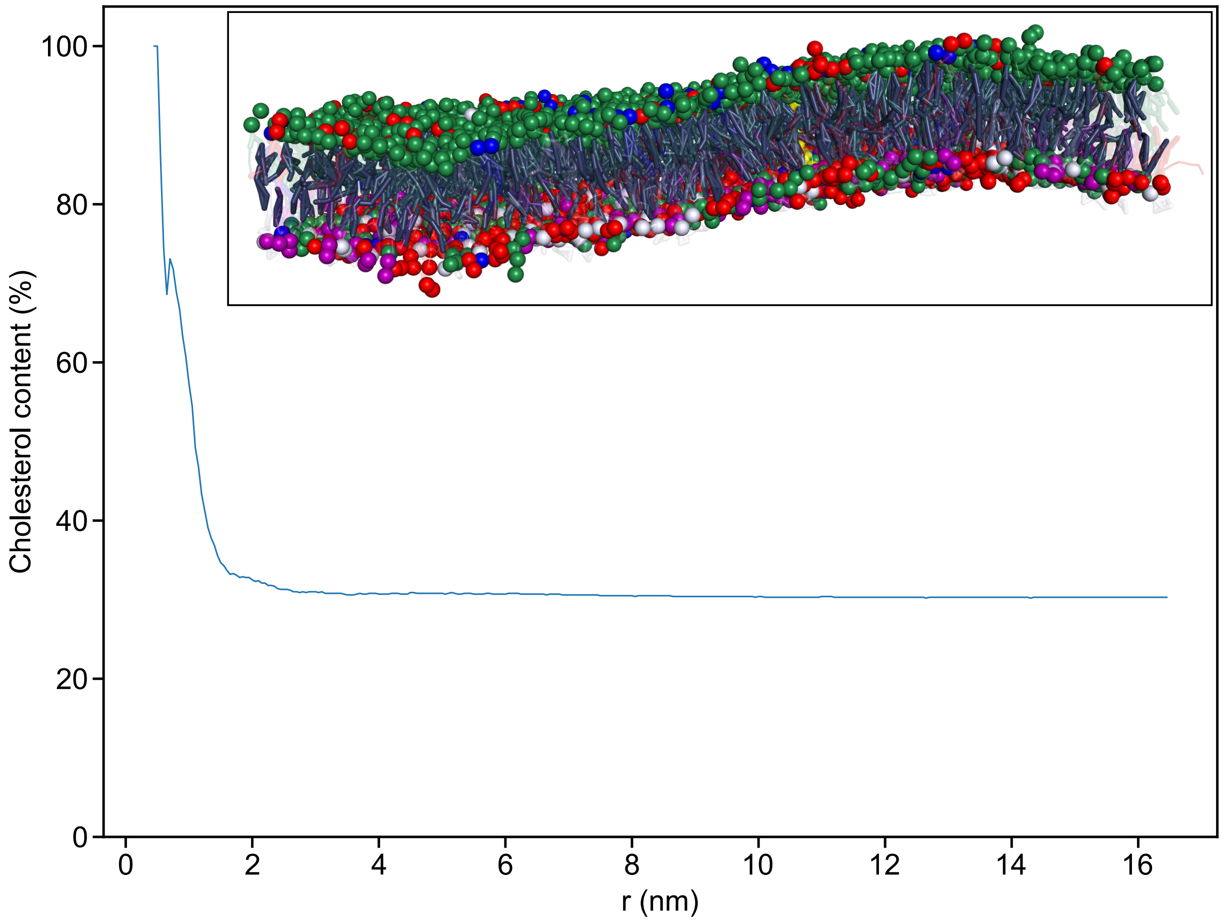

Supplementary Figure 2. A cholesterol attractor ($D_3K_3L_8K_3D_3$) produces high local cholesterol content (57.3% within 1 nm) in a native epithelial membrane model ((*1*), "Membrane 1").

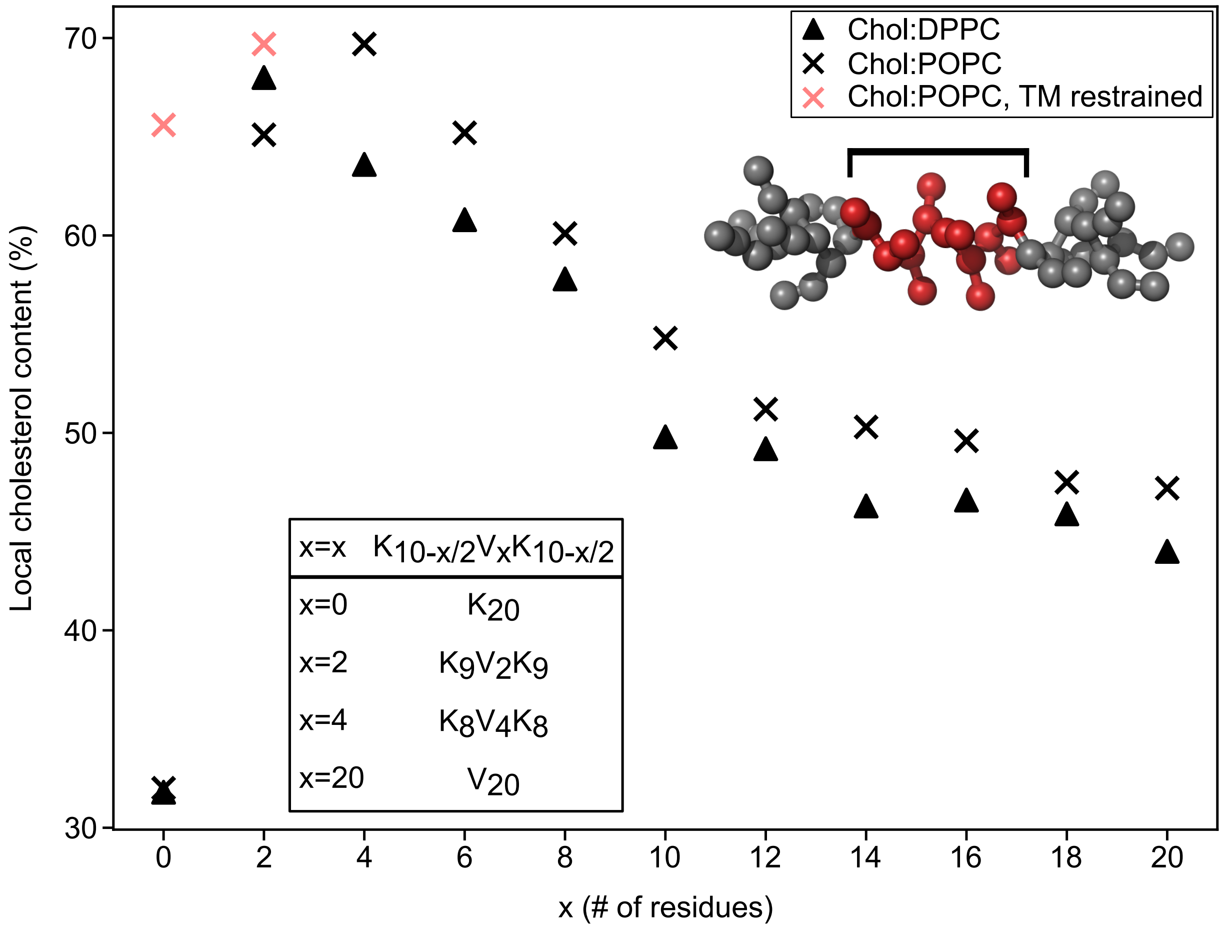

Supplementary Figure 3. **Short, hydrophobic blocks produce a high local (1.0 nm radius) cholesterol composition of the membrane.** Sequences adhere to the following motif: $K_{(10-x/2)}$–V_x–$K_{(10-x/2)}$ (x=0, 2, 4 etc.). The cholesterol attracting effect is present in both liquid-ordered (30% cholesterol, 70% DPPC) and liquid-disordered (30% cholesterol, 70% POPC) membrane phases. Restraining transmembrane position of very-short hydrophobic block sequences ($x \leq 2$) reveals high sensing functionality, persisting even in absence of the hydrophobic block (K_{20}).

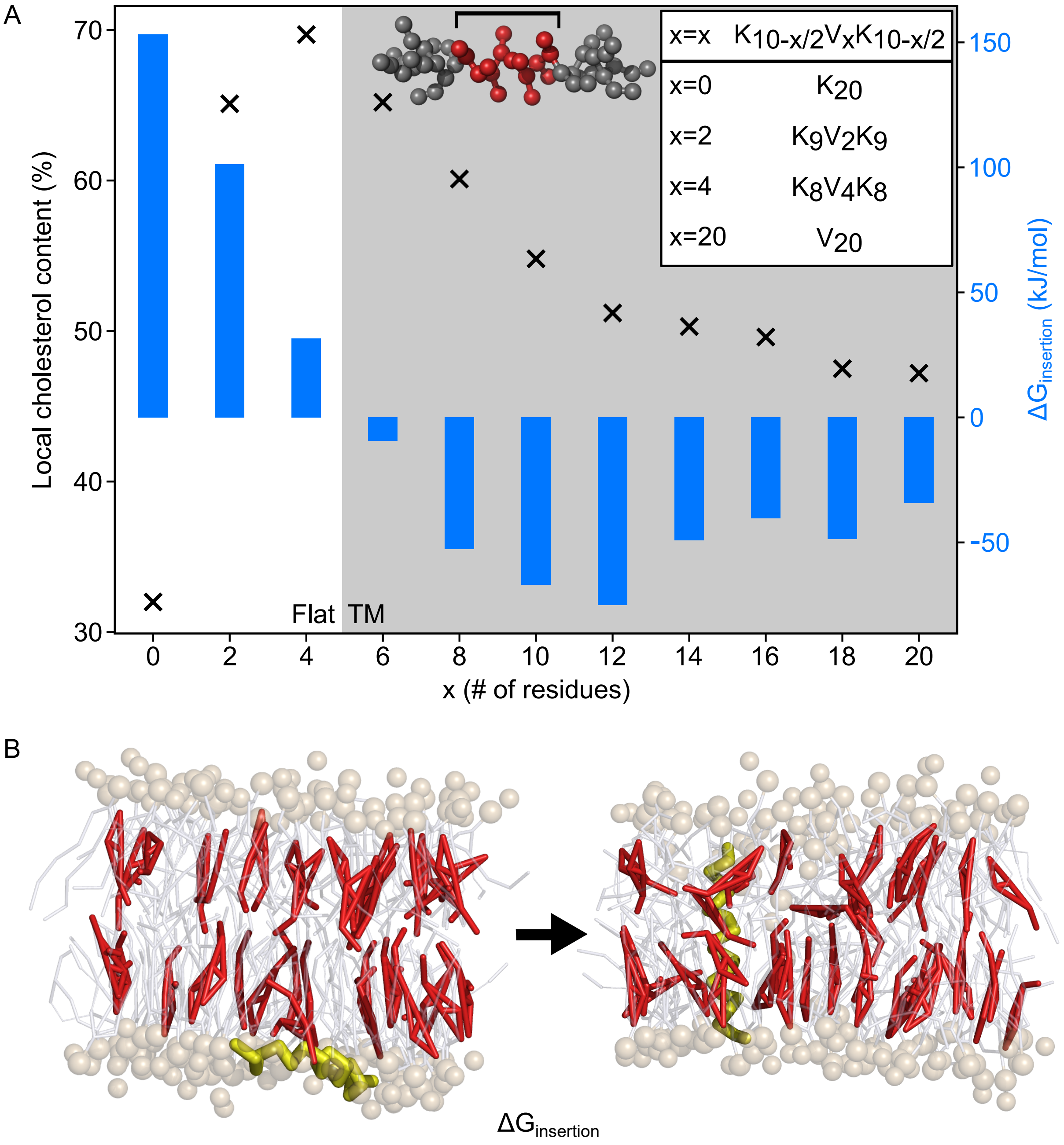

A
B
Local cholesterol content (%)
$\Delta G_{insertion}$ (kJ/mol)
x (# of residues)
Flat
TM
x=x $K_{10-x/2}V_xK_{10-x/2}$
x=0 K_{20}
x=2 $K_9V_2K_9$
x=4 $K_8V_4K_8$
x=20 V_{20}
$\Delta G_{insertion}$

Supplementary Figure 4. **The cholesterol sensing pattern follows from a trade-off between short hydrophobic block and stability of the TMD. (A):** Sequences adhere to the following motif: $K_{(10-x/2)}$–V_x–$K_{(10-x/2)}$ (x=0, 2, 4 etc.). Cholesterol attraction prefers short hydrophobic blocks, while the TMD becomes unstable at very short blocks ($x \leq 4$). Optimal cholesterol attractors are therefore likely found in the TM-favoring region near metastability ($\Delta G = 0$), as is observed in the GA-determined sequence logo (6-8 hydrophobic residues). **(B):** TM stability of the peptide is judged based on the free energy of insertion, computed between state 1 (left: peptide (yellow) flat on membrane) and state 2 (right: peptide positioned within the membrane).

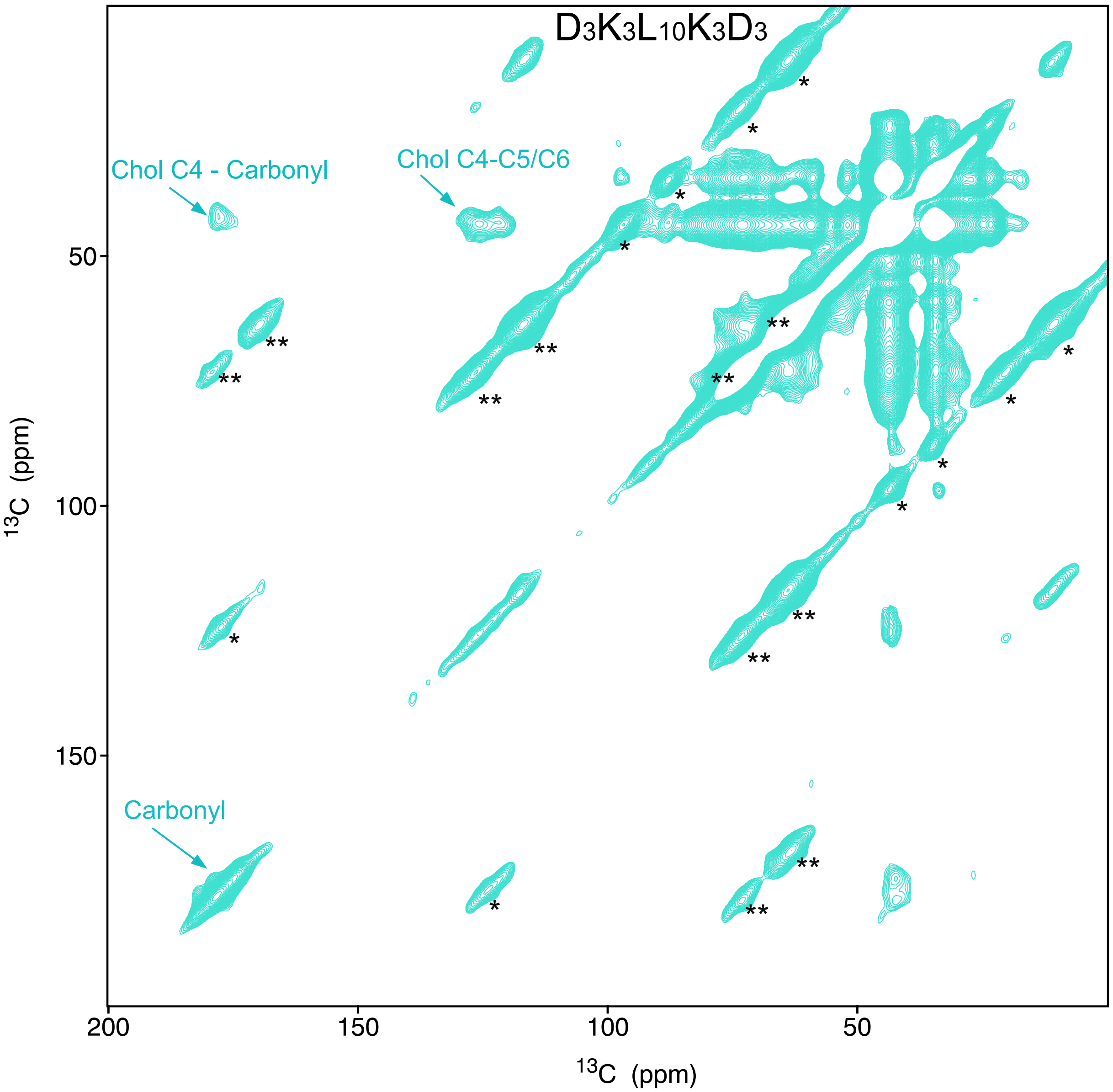

Supplementary Figure 5. 2D ^{13}C–^{13}C PDSD spectrum of L10 peptide ^{13}C-labeled at the carbonyl of the two leucine-proximal lysine residues and at C4 of cholesterol. The PDSD mixing time was 30 seconds. * side bands. ** glycerol peaks including side bands. The sample was prepared in DLPC lipid membrane.

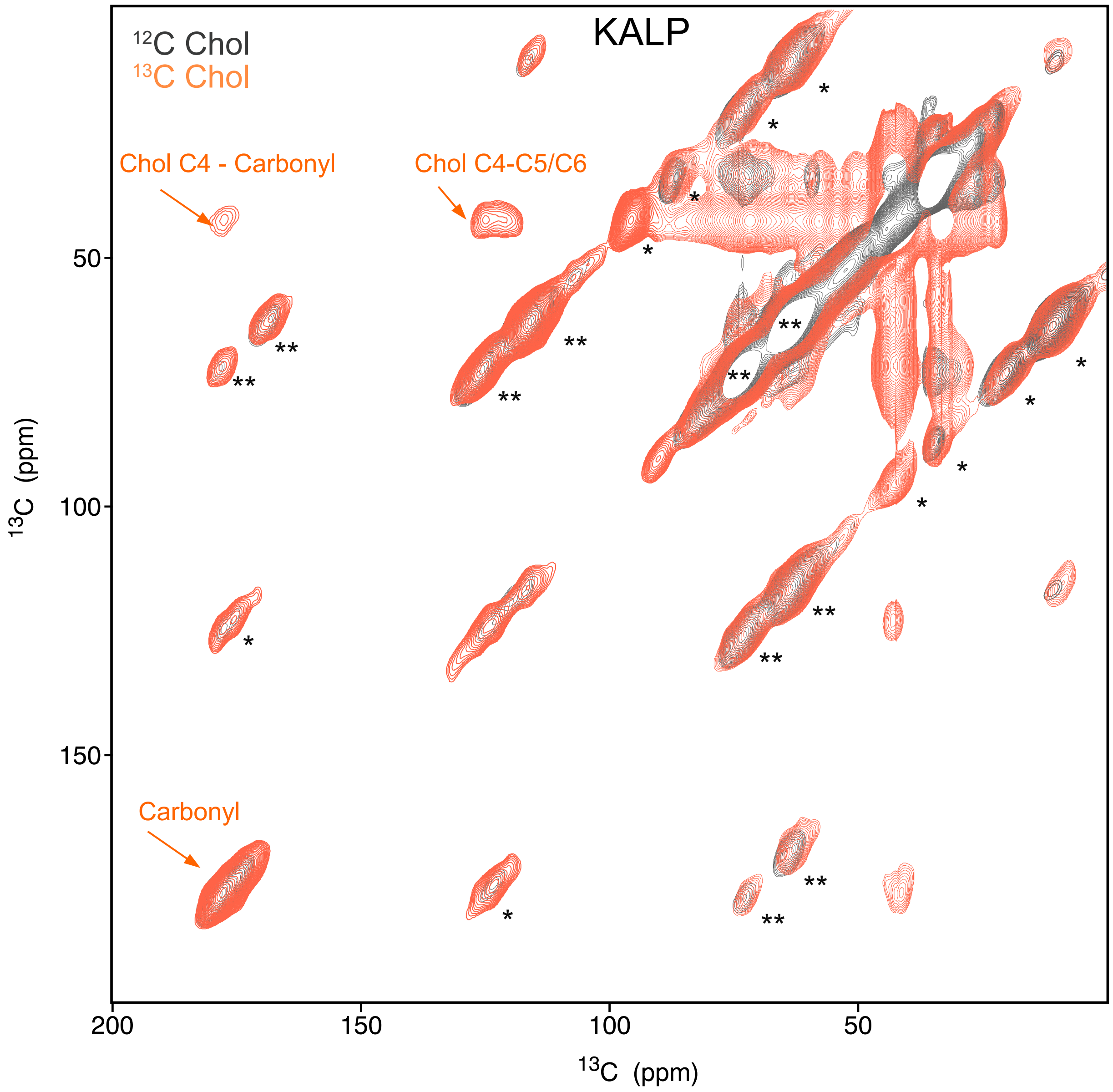

Supplementary Figure 6. 2D ^{13}C–^{13}C PDSD spectra of KALP peptide ^{13}C-labeled at the carbonyl of the two leucine-proximal lysine residues and with (red) or without (black) cholesterol ^{13}C labelling (at C4). The PDSD mixing time was 15 seconds. * side bands. ** glycerol peaks including side bands.

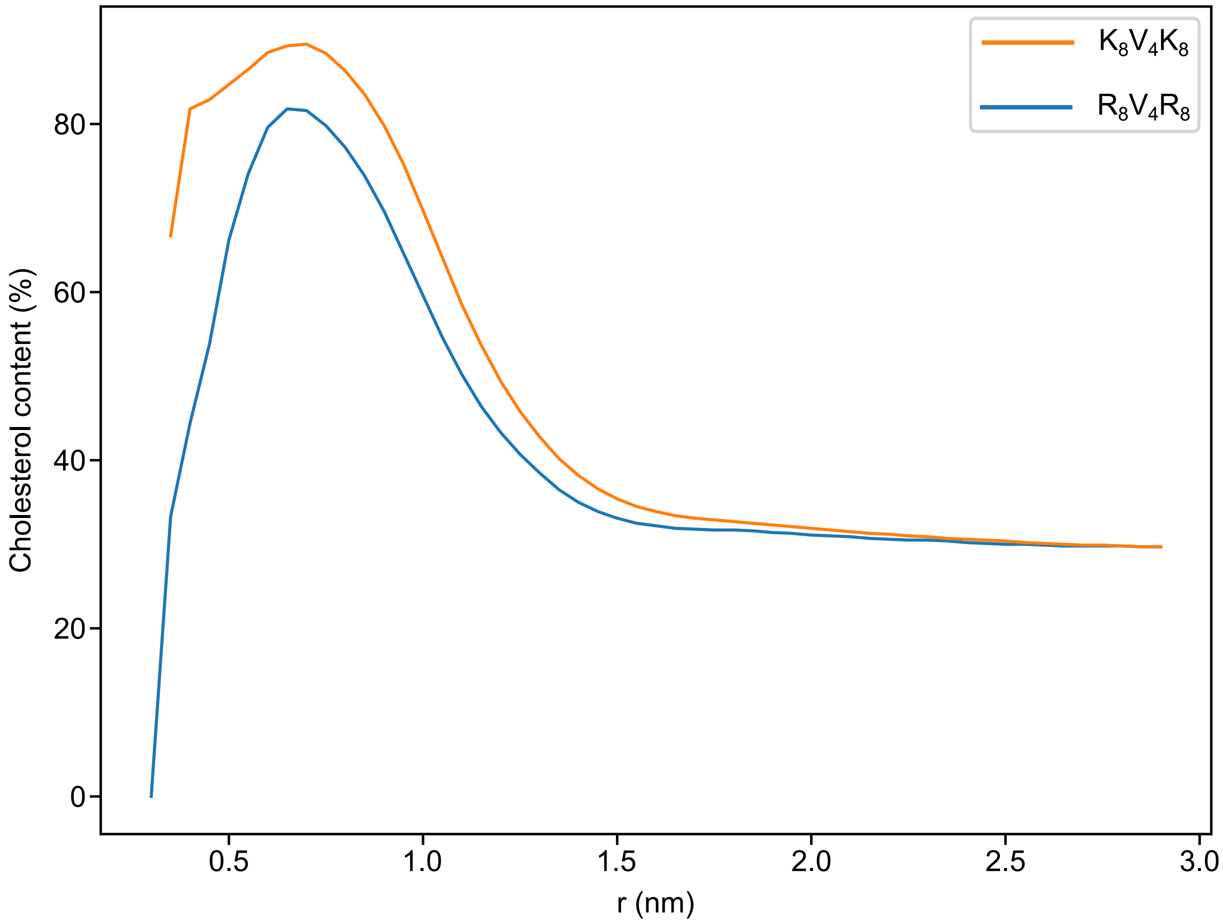

Supplementary Figure 7. Substitution of lysine (K) residues with arginine (R) residues in a dummy peptide sequence ($X_8V_4X_8$ (X=K,R)) leads to a small reduction in local membrane cholesterol content (lysine: 69.7% within 1 nm; arginine: 59.6% within 1 nm).

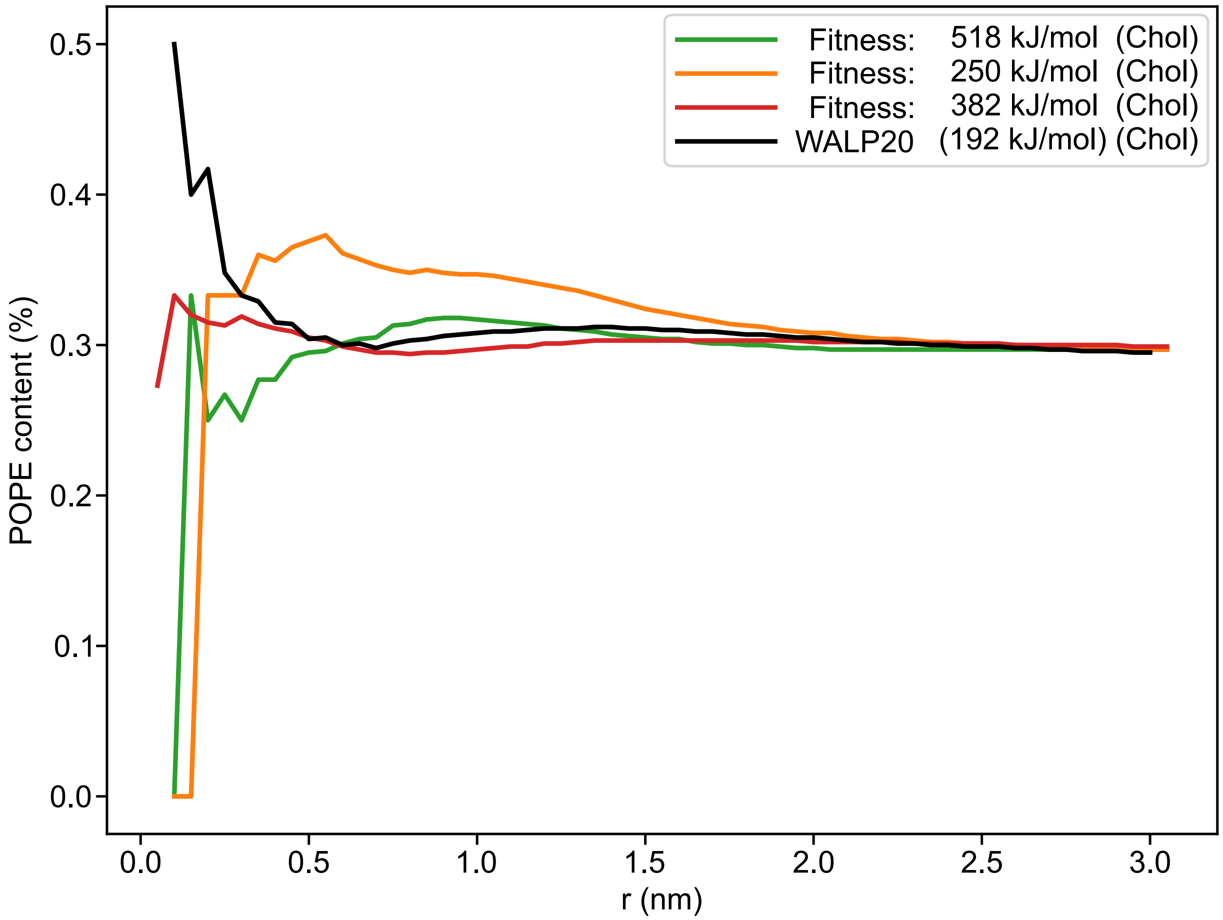

Supplementary Figure 8. The functionality of GA generated cholesterol attractors does not simultaneously correlate/translate to an increased affinity for POPE lipids in POPE/POPC (30%:70%) membranes. Ironically, the control peptide WALP20 turns out to be the best PE attractor. This illustrates that the attraction of cholesterol is mediated by a very different driving force than POPE attraction.

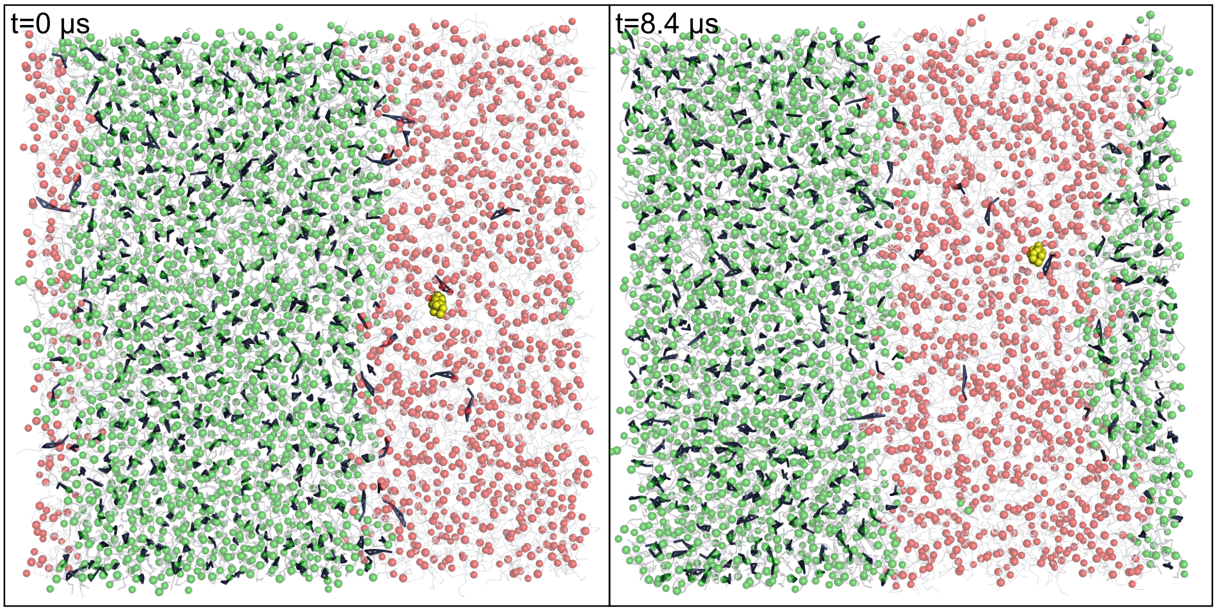

Supplementary Figure 9. A cholesterol attractor ($D_3K_3L_8K_3D_3$, in yellow) does not interact with the liquid-ordered/liquid-disordered (green/red) interface in a DPPC:DAPC:CHOL (50%:30%:20%) system. Cholesterol molecules are shown in black.

Supplementary Table 1. **Sequencing of the constructs used in the cell-based experiments.**

mScarlet-N1 10L
10L (+SG linker): **DDDKKKLLLLLLLLLLKKKDDD**SG
…CCGGTCGCCACC **ATG GAT GAC GAT AAA AAG AAG CTG TTA CTG CTT CTG TTG CTG TTA CTG CTG AAA AAG AAG GAT GAC GAT** TCC GGC GTGAG-CAAGGGC…

mEmerald-N1 11L
11L (+SG linker): **DDDKKKLLLLLLLLLLLKKKDDD**SG
…CCGGTCGCCACC **ATG GAT GAC GAT AAA AAG AAG CTG TTA CTG CTT CTG TTG CTG TTA CTG CTG CTT AAA AAG AAG GAT GAC GAT** TCC GGC GTGAG-CAAGGGC…

mEmerald-N1 KALP21
KALP21 (+ SG linker): **GKKLALALALALALALALKKA**SG
…CCGGTCGCCACC **ATG GGA AAG AAA CTG GCT CTT GCC CTG GCA TTG GCT CTG GCA CTG GCC CTG GCT CTG AAG AAG GCC** TCC GGA GTGAGCAAGGGC…

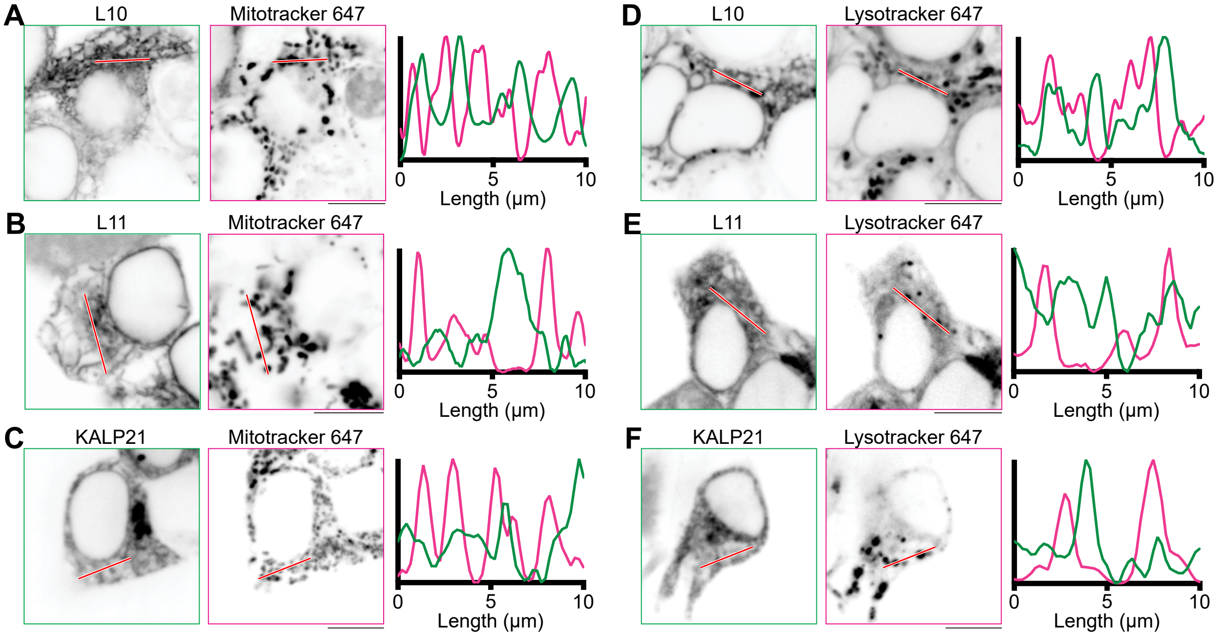

Supplementary Figure 10. L10 **(A,D)**, L11 **(B,E)**, and KALP21 **(C,F)** are distinct from the mitochondria **(A,B,C)** and lysosomes **(D,E,F)** in transfected HEK cells. For each panel, a line profile was drawn (yellow), and the normalized fluorescence intensity profiles of peptide and Mitotracker/Lysotracker are compared in the respective graphs. Scale bars and line profiles in all panels correspond to 10 μm.

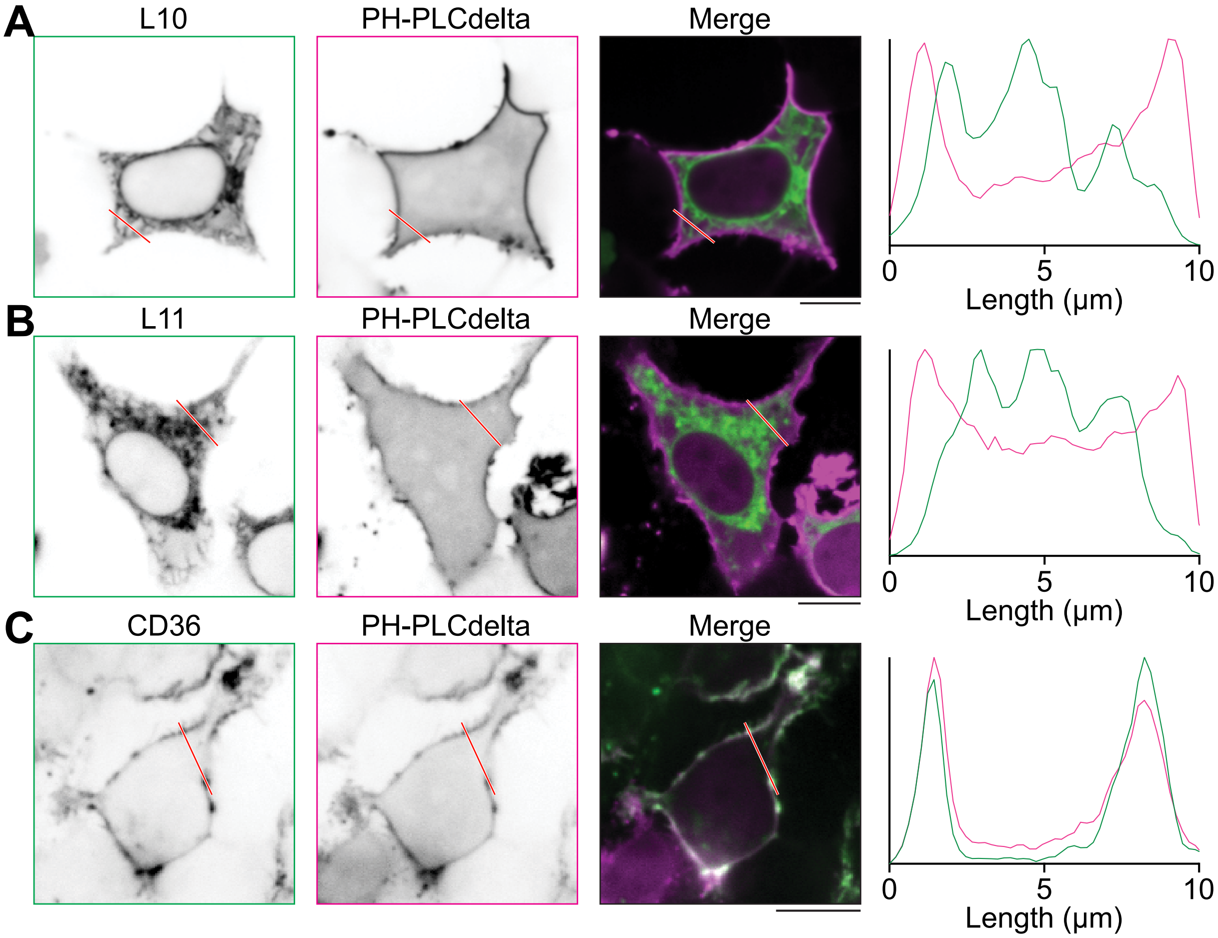

Supplementary Figure 11. L10 **(A)** and L11 **(B)** are distinct from the plasma membrane, marked using PH-PLCdelta (soluble membrane protein). **(C):** CD36 localizes to the plasma membrane. Scale bars and line profiles in all panels correspond to 10 µm.

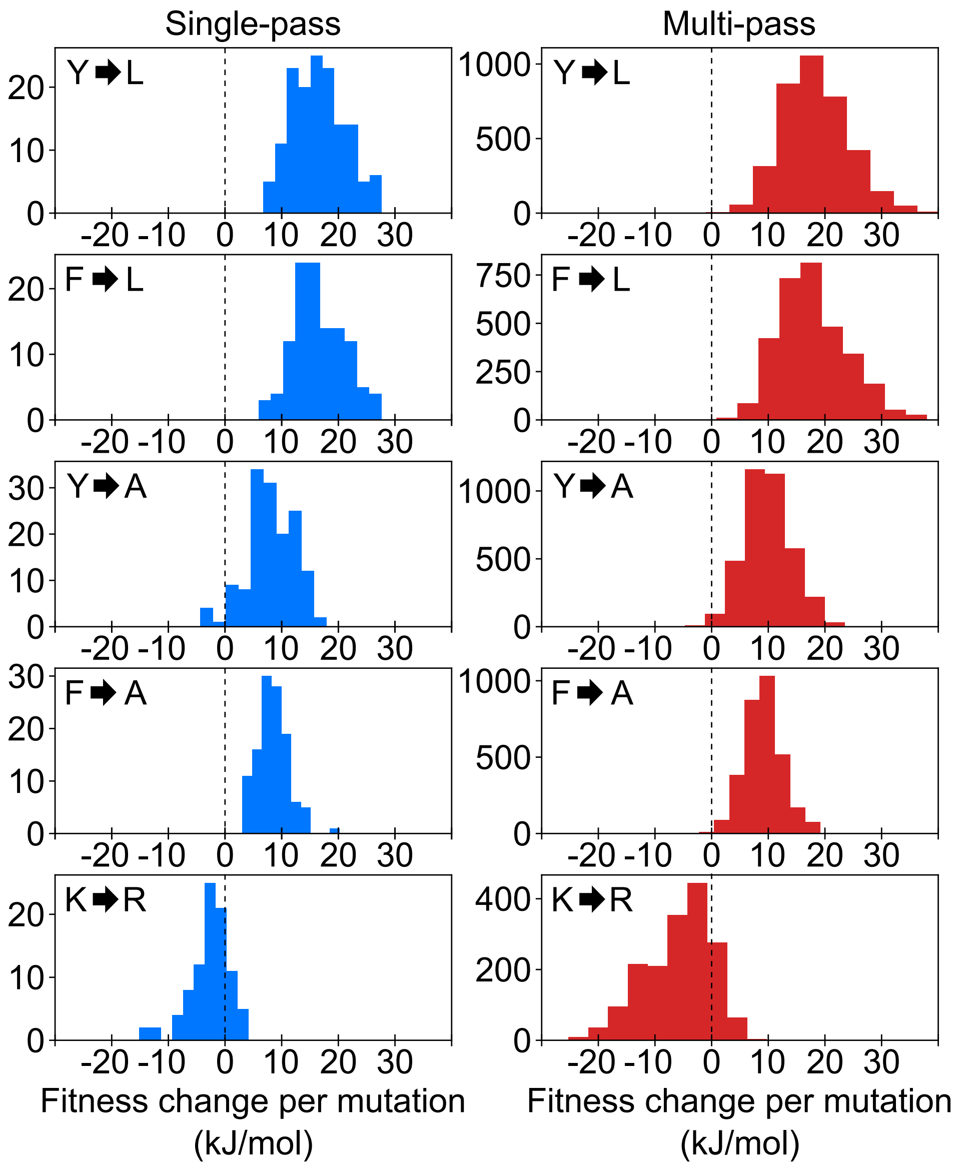

Supplementary Figure 12. Distributions of the effect of single mutations on the CNN predicted fitness of CRAC/CARC motif containing transmembrane sequences isolated from the TmAlphaFold database. Training is based on Evo-MD data generated using the Martini 2 force field.

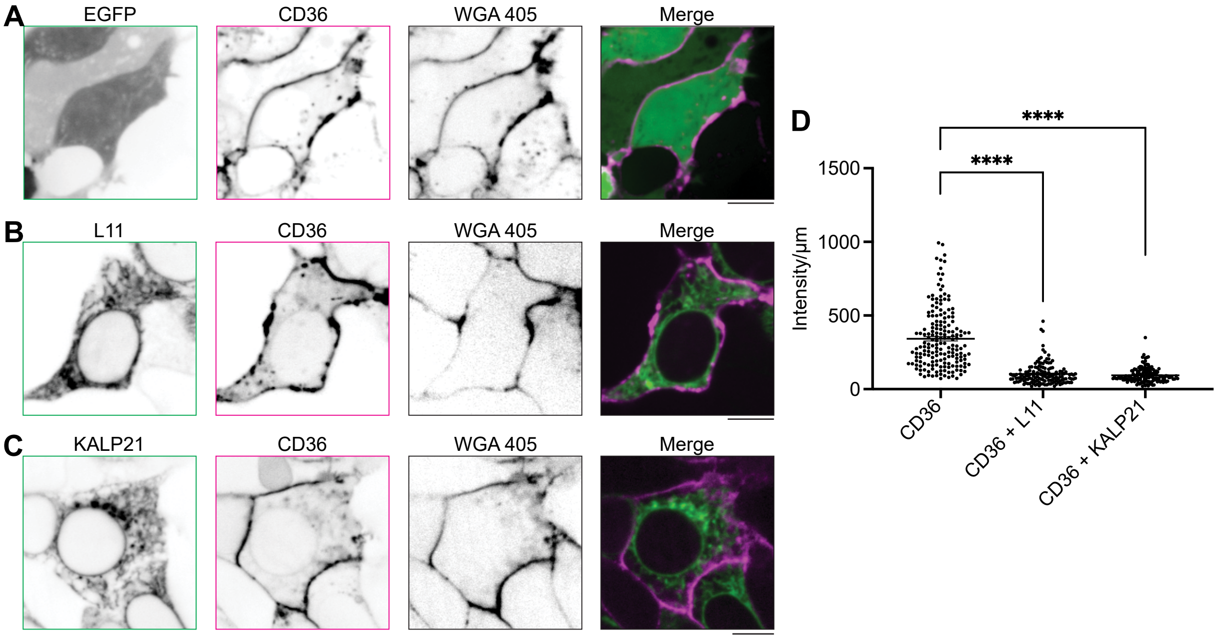

Supplementary Figure 13. L11 and KALP21 decrease the trafficking of fat transporter and scavenger receptor CD36 to the plasma membrane. This hints that overexpression of extremely short transmembrane domains which are being restricted to the ER membrane can additionally impair the expression and transport of cholesterol to distal locations. This is an additional argument on why short transmembrane domains are evolutionary not viable **(A)** Control expression of soluble EGFP and mCherry-CD36 show the major fraction of CD36 in the plasma membrane in comparison with co-expression of mCherry-CD36 with either **(B)** L11 or **(C)** KALP21 peptides. Scale bars, 10 µm. **(D)** Quantification of the average fluorescence intensity of CD36 along the plasma membrane using WGA-405 as a plasma membrane marker. (Data from three independent experiments, ****p¡0.0001, unpaired student t-test).

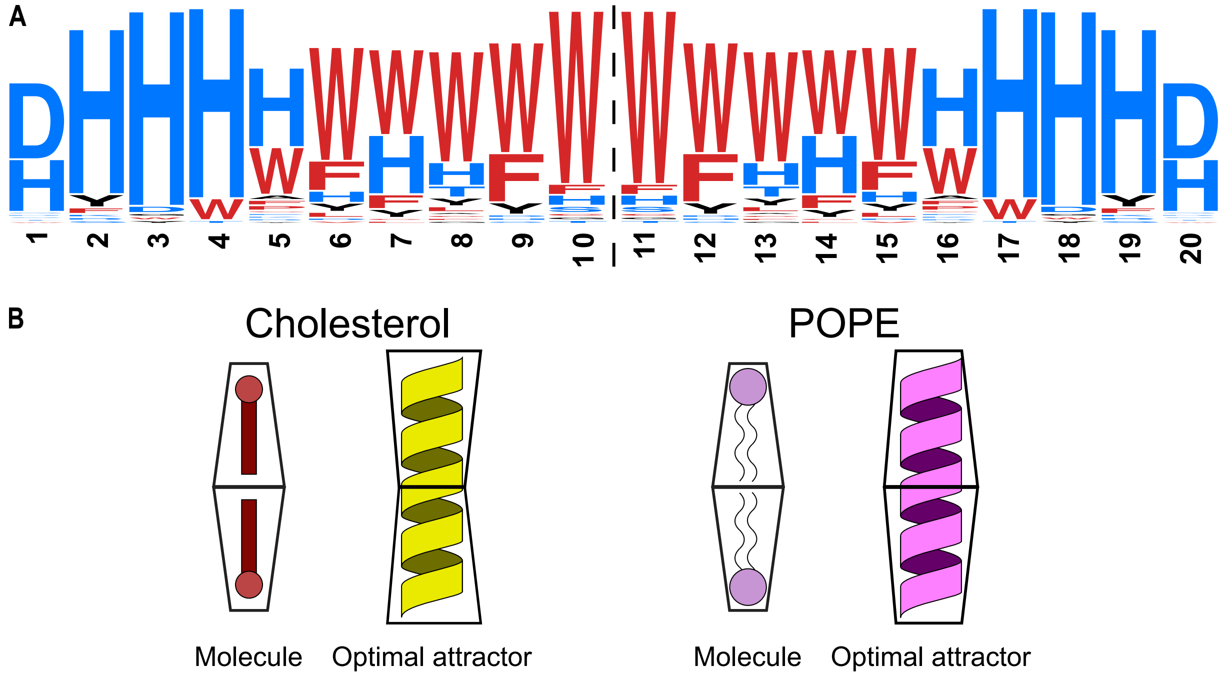

Supplementary Figure 14. **(A):** Sequence logo of a POPE attractor, computed from all high-fitness ($\geq$ 630 kJ/mol) peptide sequences, reveals a sequence distinct from the cholesterol attractor. **(B):** While cholesterol and POPE exhibit similarity in effective shape, corresponding attractor peptides vary notably in shape, suggesting a shape unspecific mechanism of attraction.

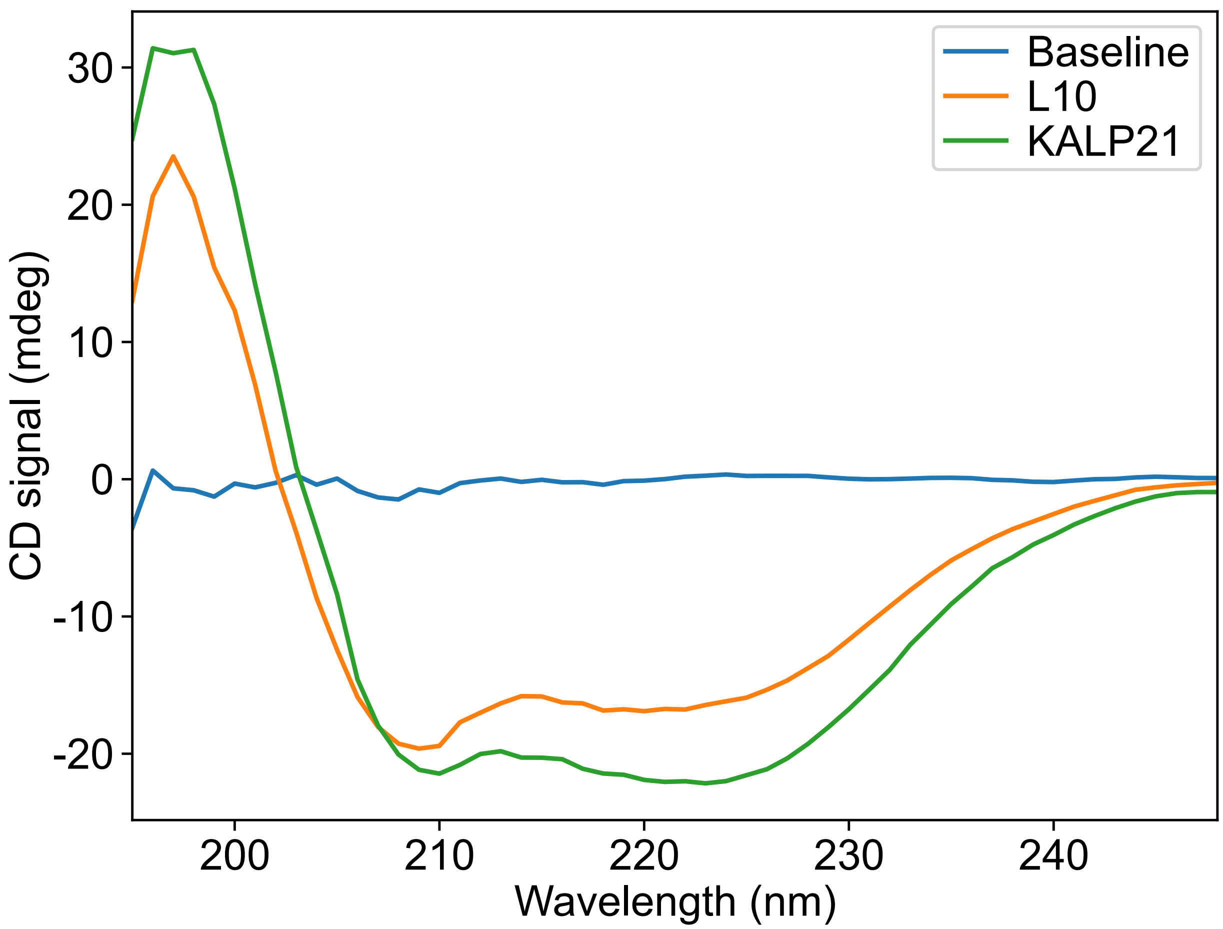

Supplementary Figure 15. Circular Dichroism (CD) measurements suggest the formation of helical structures in a membrane environment.

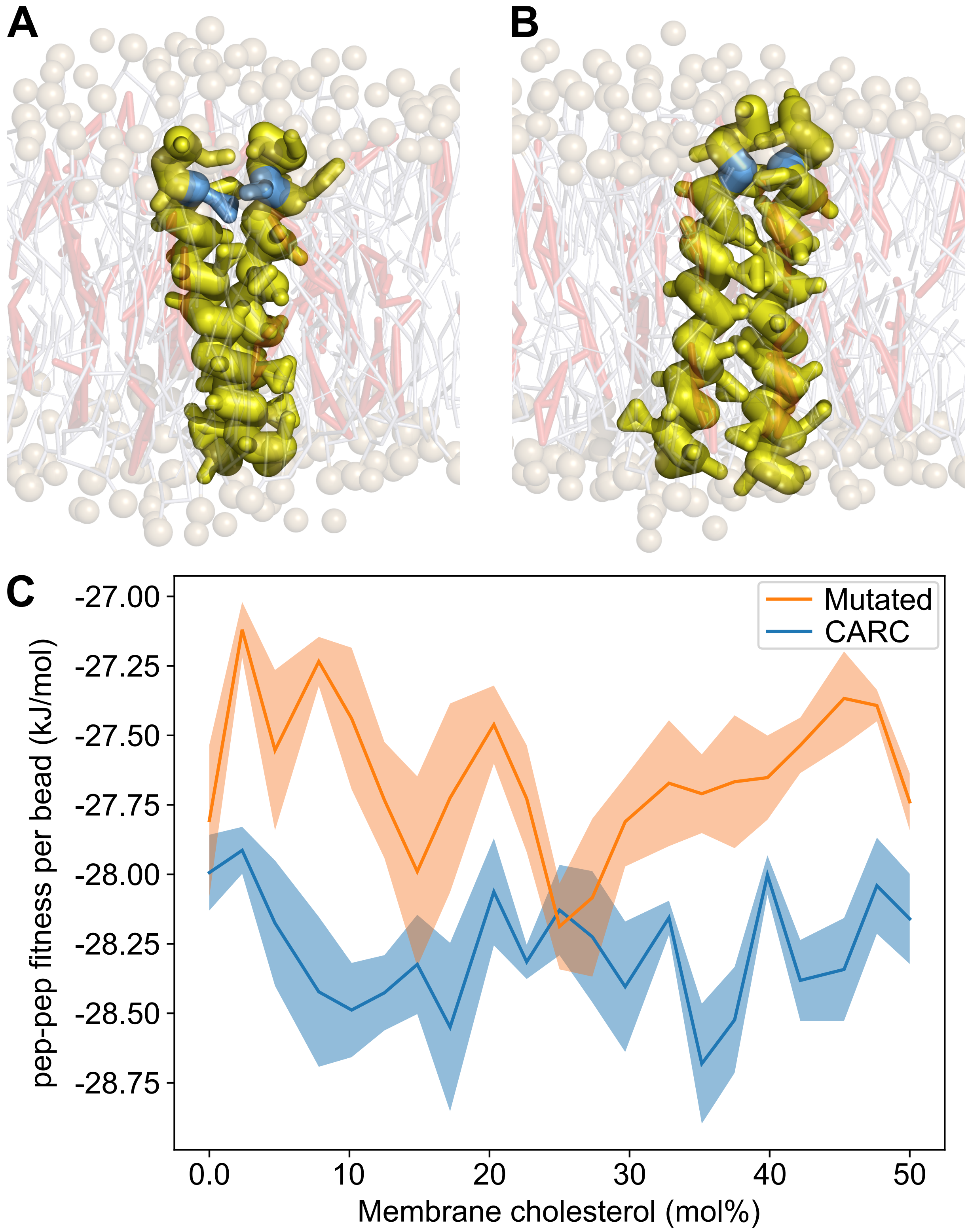
A
B
C
Mutated
CARC
-27.00
-27.25
-27.50
-27.75
-28.00
-28.25
-28.50
-28.75
pep-pep fitness per bead (kJ/mol)
0.0
10
20
30
40
50
Membrane cholesterol (mol%)

Supplementary Figure 16. Coarse-grained simulation snapshots of dimers of **(A)** the γM4 transmembrane domain, and **(B)** a 5F-A mutation thereof (residue indicated in blue). **(C)** Normalized Protein–Protein interaction energies of the dimer at varying membrane cholesterol concentrations.

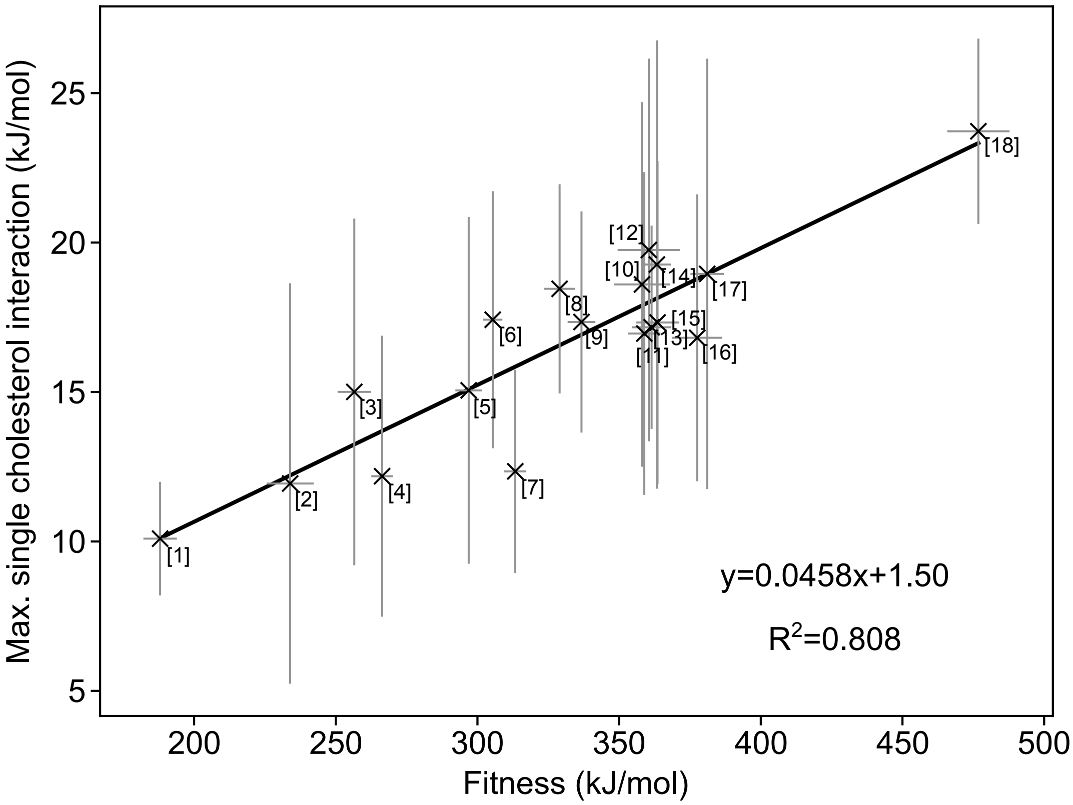

Supplementary Figure 17. Coarse-grained MD simulations of a diverse set of CRAC/CARC and cholesterol-related peptides show a correlation between total peptide–cholesterol interaction energy and the maximal single cholesterol–peptide interaction energy.

Index	Peptide	Reference
[1]	GWWLALALALALALALALWWA	WALP21
[2]	DKACFWIALLLFSIGTLAIFLTGHFNQV	Muscle nicotinic acetylcholine receptor γM4 TM domain (*2*)
[3]	DHGYTIYSTFGAFYIPLLLMLVLYGR	Serotonin 1A receptor (*3*)
[4]	HLVILGAILLALGVALTFIFRLRKGR	Human PD-L1 (*4*)
[5]	GKKLALALALALALALALKKA	KALP21
[6]	VFAFCSMLCLLNSTVNPIIYALRSK	human type-1 cannabinoid receptor (*5*)
[7]	AIVAAIVVVVVIIVTLVLIL	Best single-pass TM database hit
[8]	QRLPLYFIVNVIIPCLLFSFLTGLVFYLPT	AChR α1 (*6*)
[9]	RAGFIIYTAALGFFGPLLVICLCYLLIVVKVR	human type 3 somatostatin receptor TM5 domain (*7*)
[10]	RKPLFYIINILVPCVLISFMVNLVFYLPAD	AChR δ (*6*)
[11]	RKPLFYVINIIVPCVLISGLVLLAYFLPAQ	AChR ε (*6*)
[12]	RKPLFYLVNVIAPCILITLLAIFVFYLPPD	AChR β1 (*6*)
[13]	RLPLFYTINLIIPCLLISCLTVLVFYLPSE	AChR α4 (*6*)
[14]	DAVLILLLIPLKDKLVDPIL	Best multi-pass TM database hit
[15]	RRRTLYYGLNLLIPCVLISALALLVFLLPA	AChR α7 (*6*)
[16]	RKPLFYVINIIAPCVLISSVAILIHFLPAK	AChR γ (*6*)
[17]	RAGFIIYTAALGFFGPLLVICLCVLLIVVKVR	Y24V mutant of [9]
[18]	DDDKKKLLLLLLLLLLLKKKDDD	L11 optimal cholesterol attractor

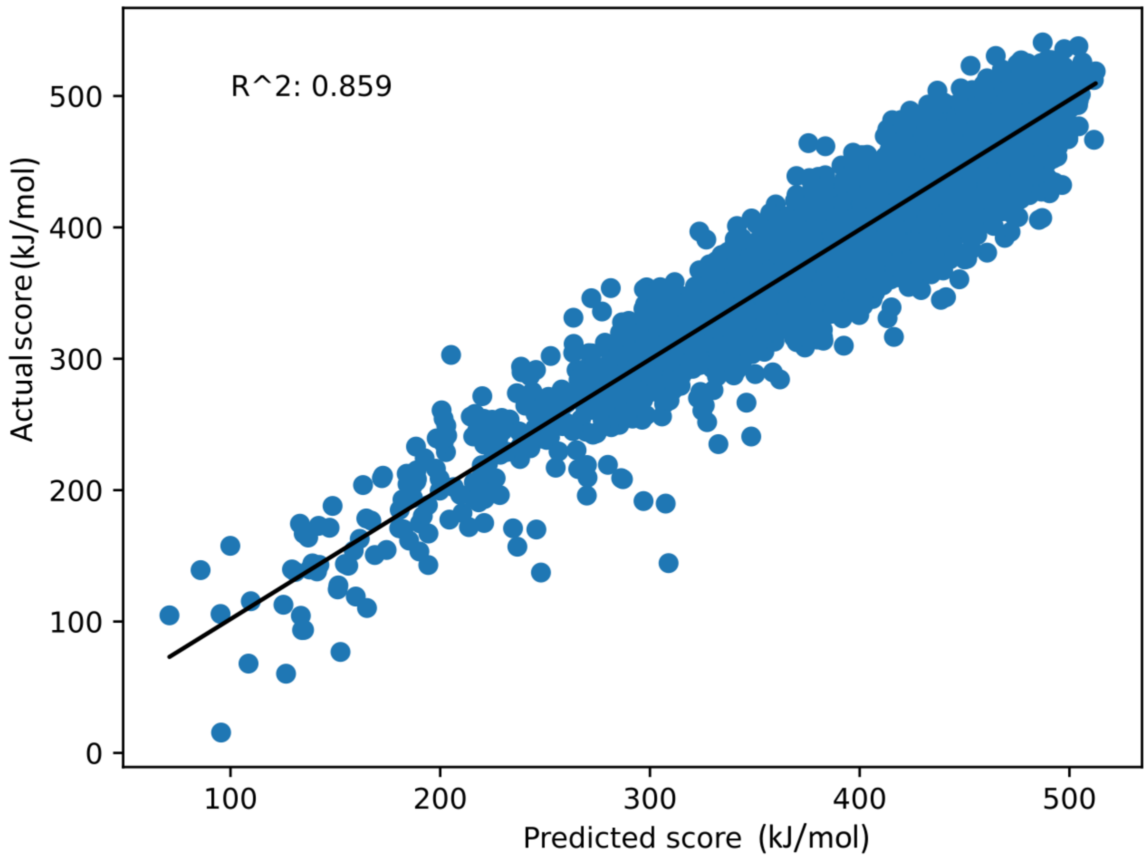

Supplementary Figure 18. Benchmark of the CNN model against molecular dynamics simulations in the prediction of TMD-cholesterol interaction enthalpy in kJ/mol.

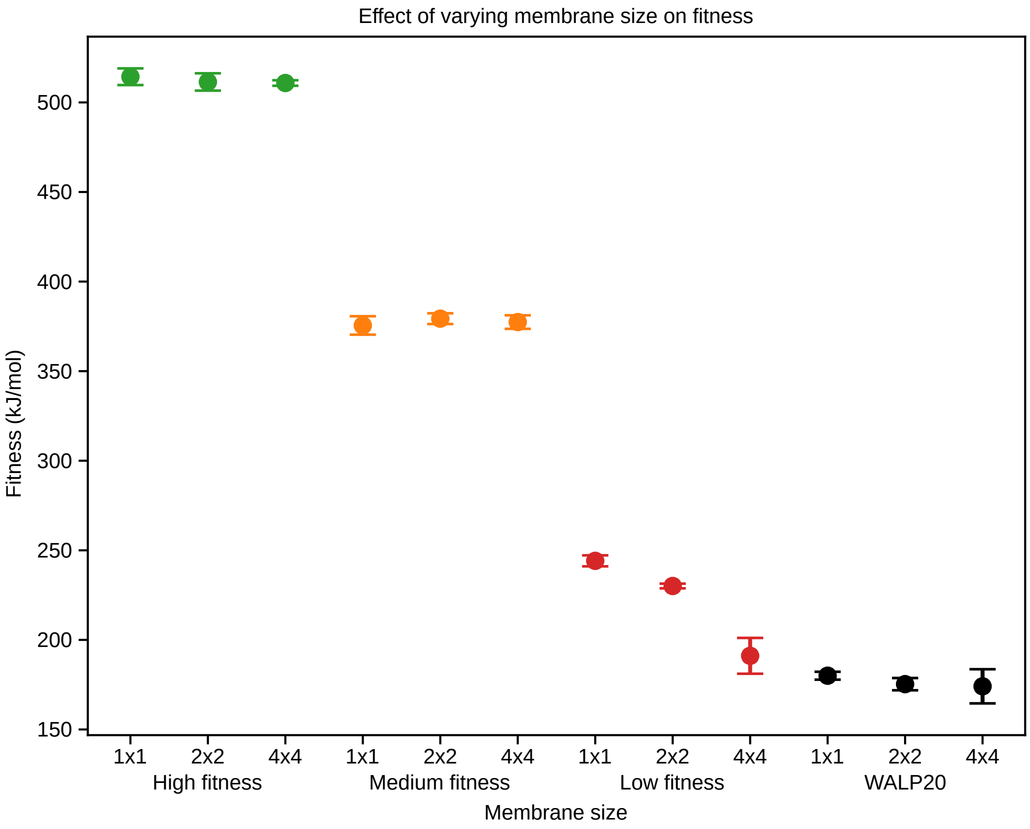

Supplementary Figure 19. Effect of membrane size on fitness. 1x1 represents the membrane size used during evolution (around 5.8 nm by 5.8 nm). 2x2 and 4x4 are larger variants of the membrane (respectively 11.4 nm by 11.4 nm and 22.7 nm by 22.7 nm). For each peptide/membrane combination, 3 independent simulations were performed. The fitness value seems to be independent of the membrane size as long as peptides are stable in the transmembrane position (”high fitness”, ”medium fitness”, and ”WALP20”). For unstable transmembrane peptides (”Low fitness”), small membrane sizes seem to stabilize the peptide’s transmembrane position, resulting in a higher fitness score compared to the larger membrane.

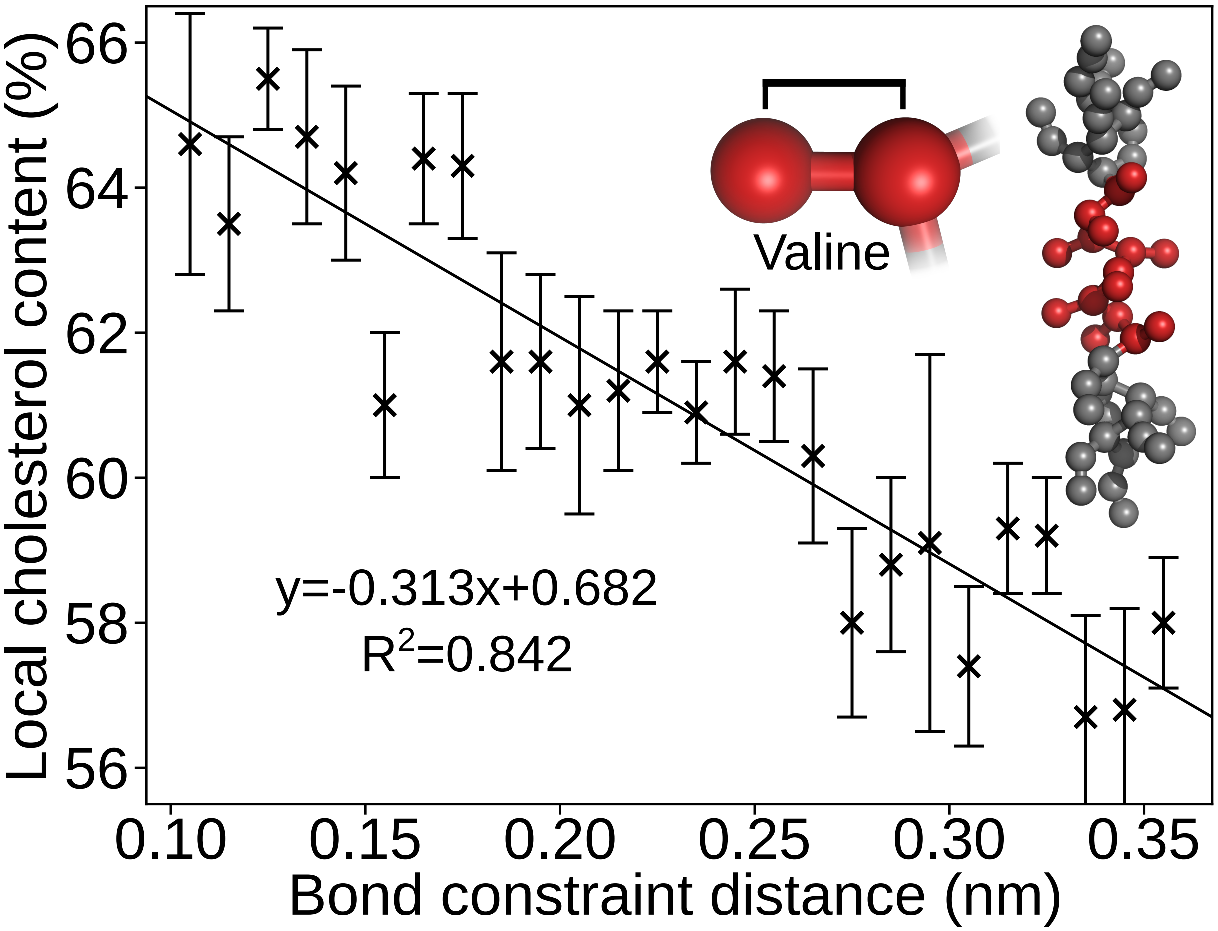

Supplementary Figure 20. The cross-sectional thickness of proteins markedly affects the local cholesterol content (defined at a 1.0 nm radius) of the membrane surrounding the protein. The force field was adapted to vary the distance between valine backbone and side chain beads in a dummy peptide (sequence: $K_6V_8K_6$). Bars represent the standard error of the mean.

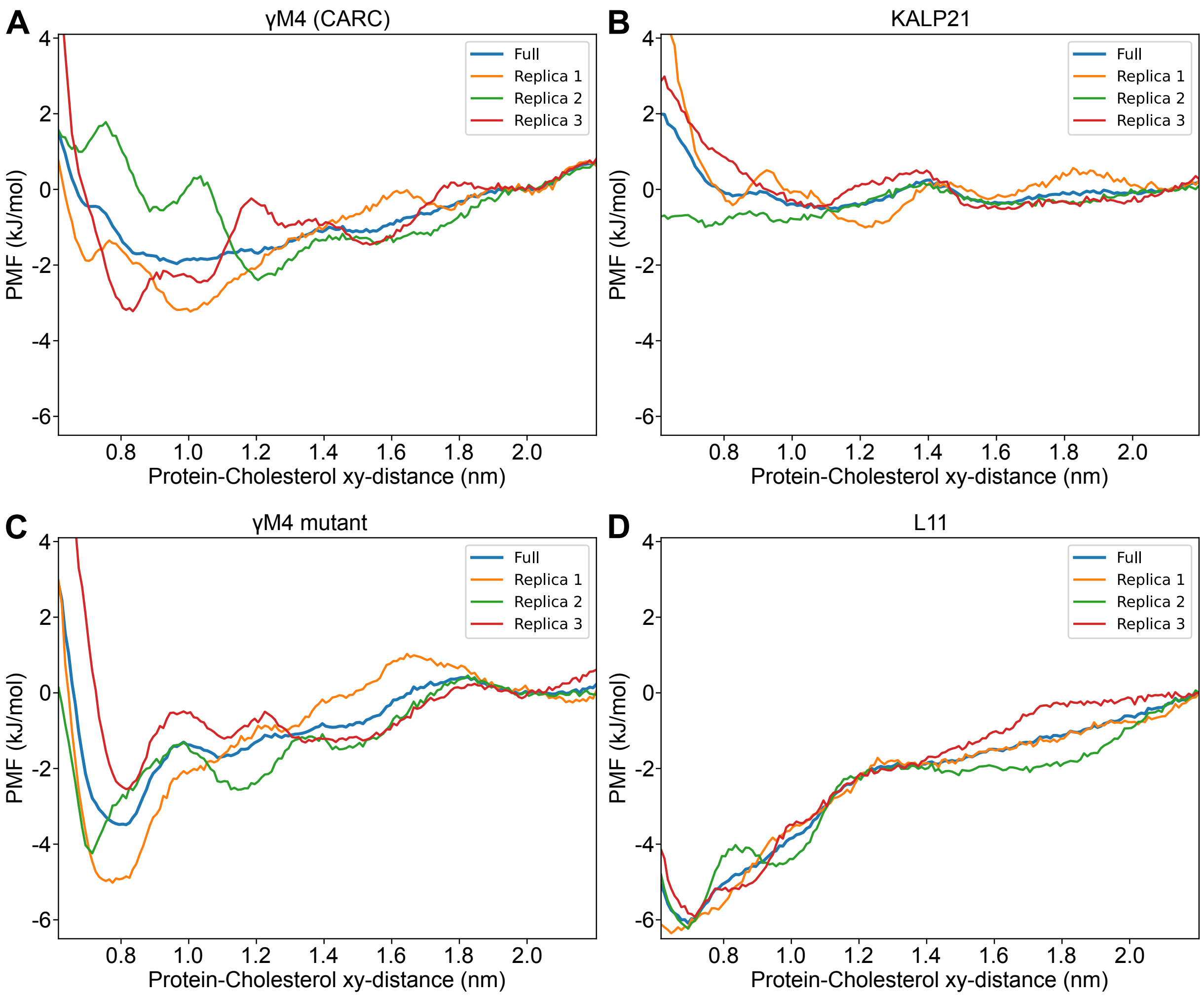

Supplementary Figure 21. Free energy profiles over the peptide-cholesterol distance computed in all-atom simulations for **(A)** CARC-containing γM4 peptide and **(C)** its non-CARC (F→A) mutant; **(B)** the stereotypical transmembrane peptide $GK_2[LA]_7LK_2A$ (KALP21), and **(D)** the rationally designed motif $D_3K_3L_{11}K_3D_3$ (L11).

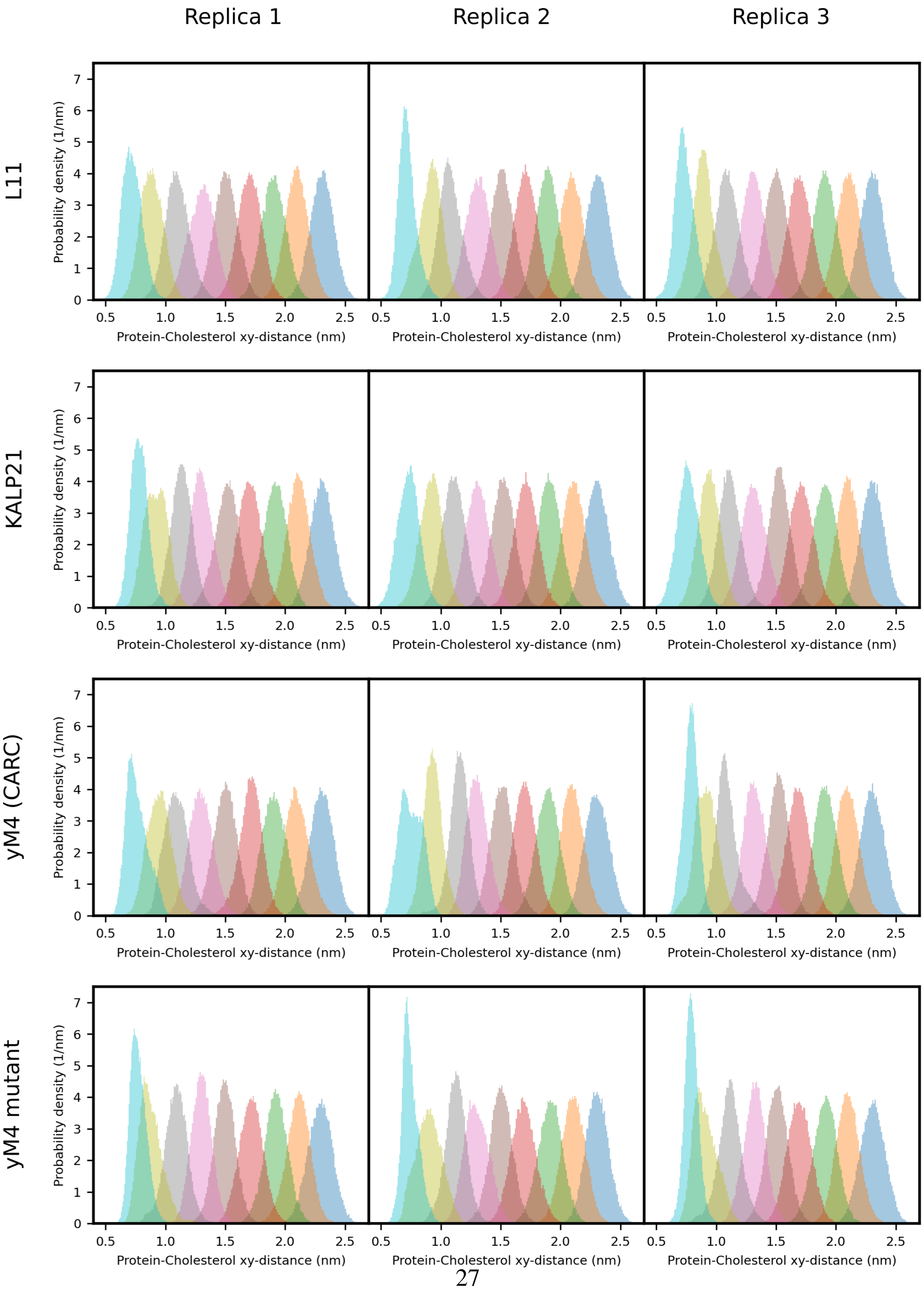
Replica 1
Replica 2
Replica 3
L11
KALP21
yM4 (CARC)
yM4 mutant
Probability density (1/nm)
Protein-Cholesterol xy-distance (nm)

Supplementary Figure 22. Sets of histograms from umbrella sampling simulations used to determine the free energy changes associated with cholesterol binding to one of the four peptides (as labeled on the left). For each system, three independent replicas were performed, each consisting of 9 umbrella sampling (US) windows (1.5 us each). The histograms display the xy-distance between cholesterol and the peptide, with colors corresponding to the individual US windows.

References

1. Wilson, K. A. *et al.* The role of plasmalogens, forssman lipids, and sphingolipid hydroxylation in modulating the biophysical properties of the epithelial plasma membrane. *The Journal of Chemical Physics* **154**, 095101 (2021).

2. de Almeida, R. F. *et al.* Cholesterol modulates the organization of the m4 transmembrane domain of the muscle nicotinic acetylcholine receptor. *Biophysical Journal* **86**, 2261–2272 (2004).

3. Sengupta, D. & Chattopadhyay, A. Identification of cholesterol binding sites in the serotonin1a receptor. *J. Phys. Chem. B* **116**, 12991–12996 (2012).

4. Wang, Q. *et al.* Regulation of PD-l1 through direct binding of cholesterol to CRAC motifs. *Sci. Adv.* **8** (2022).

5. Oddi, S. *et al.* Functional characterization of putative cholesterol binding sequence (CRAC) in human type-1 cannabinoid receptor. *Journal of Neurochemistry* **116**, 858–865 (2011).

6. Baier, C. J., Fantini, J. & Barrantes, F. J. Disclosure of cholesterol recognition motifs in transmembrane domains of the human nicotinic acetylcholine receptor. *Scientific Reports* **1** (2011).

7. Fantini, J. & Barrantes, F. J. How cholesterol interacts with membrane proteins: an exploration of cholesterol-binding sites including CRAC, CARC, and tilted domains. *Front. Physiol.* **4** (2013).
